# The effects of dopamine receptor 2 expression on B cells on bone metabolism and TNF-α levels in rheumatoid arthritis

**DOI:** 10.1186/s12891-016-1220-7

**Published:** 2016-08-19

**Authors:** Lei Wei, Ying Sun, Xiu-Fang Kong, Chi Zhang, Tao Yue, Qi Zhu, Dong-Yi He, Lin-Di Jiang

**Affiliations:** 1 Department of Rheumatology, Zhongshan Hospital, Fudan University, No. 180, Road Fenglin, Shanghai, 200032 People’s Republic of China; 2 Department of Orthopedics, Zhongshan Hospital, Fudan University, Shanghai, China; 3 Department of Rheumatology, Guanghua Integrative Medicine Hospital, Shanghai, China; 4 Center of evidence based medicine, Fudan University, Shanghai, China

**Keywords:** B cells, Dopamine receptor, Rheumatoid arthritis, Bone metabolism

## Abstract

**Background:**

Dopamine receptor 2 (DR2) expressions on B cells from Rheumatoid arthritis (RA) patients has been found to be negatively correlated with disease activity and can potentially predict the response to treatment. This study aimed to investigate the role of B cell DR2 expression on bone remodeling in RA.

**Methods:**

Patients with RA (*n* = 14) or osteoarthritis (OA; *n* = 12), and healthy controls (*n* = 12) were recruited for this study. Dopamine receptor (DR) 2 expression was assessed using flow cytometry. Pro-inflammatory cytokines, including interleuin(IL)-1β, IL-6, IL-17, and tumor necrosis factor(TNF)-α, and bone turnovers, including osteocalcin (OC),serum procollagen type I N propeptide (PINP), C-terminal telopeptide of type I collagen (β-CTX), collagen type I cross-linked telopeptide (ICTP), as well as matrix metalloproteinase-3 (MMP-3) and osteoprotegerin (OPG) were measured by electrochemiluminescence, chemiluminescence, or enzyme-linked immunosorbent assay. DR2 expression on synovial B cells from 4 RA patients and 3 OA patients was detected by immunofluorescence.

**Results:**

There were more DR2^+^CD19^+^ B cells in synovial tissues from RA patients than in those from OA patients. The frequency of peripheral B cells that expressed DR2 was positively correlated with plasma TNF-α level. Levels of ICTP and MMP-3 were significantly higher, and OPG were lower in RA patients compared to those in the OA group and healthy controls (all *P* < 0.05).

**Conclusion:**

The frequency of B cells that expressed DR2 showed a correlation with levels of the pro-inflammatory cytokine TNF-α. DR2^+^CD19^+^ B cells in synovial tissues might have a role in bone metabolism and TNF-α production.

## Background

Accumulating evidence has demonstrated that the immune and skeletal systems share an abundance of molecules and regulatory mechanisms, including cytokines, receptors, signaling molecules, and transcription factors, are the focus of the field of osteoimmunology [1]. The dynamic homeostasis of bone formation and resorption can be disrupted in many autoimmune disorders because dysregulated immune responses can trigger inflammation and accelerate the activation of osteoclastogenesis. As a prototypical immune disease, rheumatoid arthritis (RA) is a type of chronic inflammatory arthritis characterized by juxta-articular bone destruction and systemic osteoporosis [2], which is closely related to the course of disease and disability. B cells, which play a key role in humoral immunity, are one of the most important cell types in osteoimmunology. B cells are uniquely endowed with osteointeractive properties, as they can induce osteoclastogenic effects by secreting inflammatory cytokines or autoantibodies, directly activate osteoclasts via cell-cell contact, or potentially act as the precursors of osteoclasts [3].

Reciprocal effects have also been observed between the neurological and immune systems. These connections have been extensively studied and are the focus of the interdisciplinary field of neuroimmunology [4]. The dopaminergic system, which consists of dopamine and dopamine receptors (DRs), is a critical neurological pathway that can modulate the immune system [5]. Dopamine targets one of five receptors, namely DR1–DR5, to produce distinct effects on the survival, differentiation, proliferation, polarization, and apoptosis of immune cells, as well as inducing different cytokine profiles. Altered expression of dopamine receptors has been observed in many autoimmune disorders [6].

Interestingly, the dopaminergic system also has tight relationship with the skeletal system. Increased dopamine receptor expression has been observed in synovial fibroblasts in RA [7]. Genetic polymorphisms in DR4 have been significantly associated with bone mineral density in Japanese men [8]. Neuroleptic treatment in Parkinson’s disease using a D2-like receptor antagonist has been reported to lead to osteoporosis [9]. Additionally, activation of the D2-like receptor inhibits osteoclastogenesis by directly affecting osteoclast formation, and antagonizing the D1-like receptor pathway to suppress bone and cartilage destruction in both mice with collagen-induced arthritis [10] and in humanized RA/SCID mice [11].

Previously, we found that DR2 expression in B cells is negatively correlated with disease activity in RA patients and can reflect patient responses to disease modifying antirheumatic drug therapy [12]. As the immune, bone, and dopaminergic systems are interconnected, we sought to further investigate the relationships between these systems and to gain a better understanding of the pathogenesis of RA. Herein, bone turnovers and inflammatory cytokine levels were measured in patients with RA at baseline and after drug therapy, and were compared with the frequency of B cell DR2 expression. We also measured B cell DR2 expression at the bone–synovial interface. Since the DR2 expressed on the B cells might play a pivotal role in the generation and amplification of focal inflammation.

## Methods

### Study population

A total of 14 RA patients were included in this study and all patients satisfied the 1987 American College of Rheumatology classification criteria for RA [13]. Additionally, 12 healthy volunteers and osteoarthritis (OA) patients diagnosed according to the classical classification criteria [14] were recruited as controls. Inclusion criteria for the RA group included patients with active disease (28-joint disease activity score [DAS28] according to the CRP formula > 3.2) and disease modifying anti-rheumatic drug (DMARD) naive or no DMARDs use within the previous 3 months. Patients who had previously received anti-tumor necrosis factor-α (TNF-α) or glucocorticoid therapy were excluded. The other exclusion criteria for both the RA and OA groups were the followings: infectious or inflammatory disease, an endocrine disorder, any past or current psychiatric or neurological diseases, pregnant or planning to be pregnant, lactation, liver or kidney dysfunction, cardiovascular disease, cancer, any drug history that would affect the sympathomimetric or sympatholytic system, and recent severe stress events. This study was performed according to the Declaration of Helsinki and approved by the Medical Ethics Committee of the Zhongshan Hospital. All patients provided written informed consent.

### Sample preparations

Anti-coagulating peripheral blood (10 ml) from the cubital vein was obtained before (i.e., at baseline) and 3 months after the initiation of treatment. Peripheral blood mononuclear cells were separated using Ficoll–Hypaque density centrifugation and prepared at a final concentration of 1 × 10^6^ cells/ml. Plasma was separated and stored at −80 °C. Synovial tissue samples were obtained from another 4 patients with RA and 3 patients with OA who had been diagnosed according to the classic classification criteria and undergone knee joint replacement surgery. Fat tissue was carefully removed from the synovial tissue. Dissected samples were immediately fixed in O.C.T. (Sakura Finetek, Japan), quickly frozen, and stored at −80 °C until later use.

### DR2 detection on peripheral B cells by flow cytometry

B cells from PBMCs were labeled with anti-human allophycocyanin (APC)-conjugated CD19 antibody (Becton Dickinson, San Jose, CA, USA) and DR2 antibody (Life Span BioSciences, Seattle, WA, USA) at 4 °C for 30 min. Cells were washed twice with staining buffer and incubated with secondary CF405M-conjugated goat anti-rabbit antibody (Sigma–Aldrich, St. Louis, MO, USA; 1:100 dilution) at 4 °C for 30 min. A ‘no primary antibody’ control and a normal rabbit sera (Rab) control (1:100) were used as two separate negative controls. Stained cells were analyzed using a Canto II flow cytometer (BD Biosciences). Data analysis was performed with Diva software (BD Biosciences) and FlowJo V.7.6.4 (Treestar Inc., Ashland, OR, USA). A minimum of 1 × 10^6^ cells were analyzed from each sample. The results were finally expressed as the percentage of positive cells (%). BDR2 (%) indicated the percentage of CD19^+^DR2^+^ cells among total CD19^+^ cells.

### Measurement of cytokines and bone turnover

Biochemical markers of bone formation—Osteocalcin (OC) or serum procollagen type I N propeptide (PINP), and bone resorption—C-terminal telopeptide of type I collagen (β-CTX) or collagen type I cross-linked telopeptide (ICTP), as well as matrix metalloproteinase 3 (MMP3) and osteoprotegerin (OPG) were tested. Plasma OC, PINP, and β-CTX were measured using an electrochemiluminescence immunoassay with automated immunoassay analyzers (Elecsys and Cobas e601 Analyzer, 2010, Roche Diagnostics GmbH, Mannheim, Germany) and reagents were supplied by Roche Diagnostics (Roche Diagnostics GmbH). The total CV was <6 %. Plasma ICTP was measured manually using an ELISA kit from Orion Diagnostics (Orion Corporation, Orion Diagnostica, Espoo, Finland). The normal range of serum ICTP was 1.8–5.0 ng/ml. Inter-assay CVs for ICTP were 4–9 %. Quantification of levels of the pro-inflammatory cytokines interleukin-1β (IL-1β), IL-6, and TNF-α in plasma were conducted on a Siemens Immulite 1000 immunoassay platform. IL-17 and MMP3 protein expression levels were measured in plasma by using specific ELISA kits (R&D Systems Europe, Abingdon, UK). The ELISA standard curve range for IL-17 was from 15 to 1000 pg/ml. Plasma OPG was measured using a highly sensitive quantitative sandwich ELISA (Sigma–Aldrich) test. Intra- and inter-assay coefficients of variation were 2.36 % and 5.97 %, respectively.

### Immunohistochemical analysis of human synovial tissues

Immunohistochemistry was performed following standard protocols. In brief, cyosections (5 μm) were air-dried for 1 h at room temperature. After fixation with acetone for 10 min, sections were washed with phosphate-buffered saline. Nonspecific binding sites were blocked with goat serum for 60 min at 37 °C. Samples were incubated with DR2 primary antibody and APC-conjugated CD19 antibody overnight at 4 °C. The next day, sections were thoroughly washed with PBS, and then were incubated with specific Alexa Fluor 488- and Alexa Fluor 647-conjugated antibodies (Sigma–Aldrich) for 45 min. Sections were counterstained with 4′-6-Diamidino-2-phenylindole dihydrochloride (DAPI; Sigma–Aldrich). Samples were mounted with glycerol glycine and covered with a coverslip. Every antibody was tested using single staining for specificity and autofluorescence. Staining with the secondary antibodies alone was performed in parallel as negative control, and no positive staining was observed.

### Statistical analysis

Continuous data were expressed as means ± standard deviation (SD) or medians (inter-quartile range, IQR) according to the data distribution. Normal distribution of data was confirmed using the Kolmogorov–Smirnov test. Statistical significance was evaluated by Student’s *t*-test. For non-parametric data, the Mann–Whitney *U*-test was used. The Spearman’s correlation coefficient was used for correlation analyses. All statistical analyses were carried out using the Statistical Package for the Social Sciences (SPSS) V.20. *P*-values <0.05 were considered indicate statistically significant differences.

## Results

### General characteristics

**Fig. 1:**
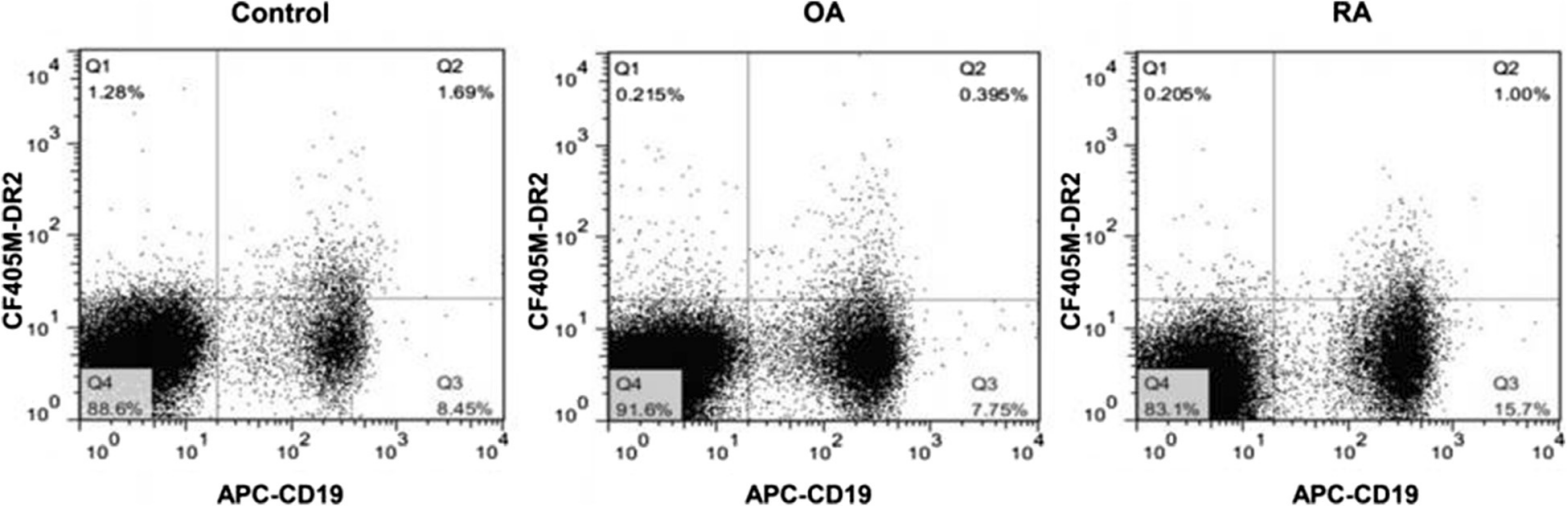
Representative FACS plots of DR2-expressing cells in patients with RA, OA and healthy controls

Among the 14 RA patients included in this analysis, 11 were female (78.57 %). The mean age of the RA patients was 54.29 years old (SD, 14.96; range, 20–77). As controls, 12 OA patients and 12 healthy donors were also recruited; their ages were 56.75 ± 9.19 and 24.58 ± 2.07 years old, with male/female ratios of 1:1 and 1:2, respectively. All RA patients had moderate to high disease activity (DAS 286.29 ± 1.15). The frequency of cells showing positive staining for DR2 in the healthy control, RA, and OA groups were 28.1 % (14.38 %–42.35 %), 3.58 % (2.58 %–8.13 %), and 6.76 % (2.44 %–15.85 %), respectively. RA patients had lower frequencies of DR2-expressing cells in contrast to the healthy controls (*P* = 0.006), and had no statistical difference with OA patients (*P* = 0.547) (Fig. 1).

### Bone turnover and inflammatory markers

Serum levels of the cytokines IL-1β, TNF-α, IL-6, and IL-17 were measured. IL-1β levels did not differ among the three groups (<5 pg/ml). IL-6 was higher at baseline in the RA group (median, 9.70 pg/ml; IQR, 7.9–71.95 pg/ml) than in the OA group (median, 2.60 pg/ml; IQR, 2.0–2.70 pg/ml; *P* < 0.001) or the healthy controls (median, 2.00 pg/ml; IQR, 2.0–2.38 pg/ml; *P* < 0.001). Levels of TNF-α in the RA group (median: 16.2 pg/ml; IQR, 12.53–24.00 pg/ml) were remarkably higher compared to the healthy controls (median, 10.45 pg/ml; IQR, 8.05–17.33 pg/ml; *P* = 0.017) and the OA group (median, 14.4 pg/ml; IQR, 11.03–20.58 pg/ml), although this difference was not statistically significant (*P* = 0.410). Levels of IL-17 were below the limit of detection in the healthy controls and OA group, and were higher than 15 pg/ml in 14 RA patients.

The levels of OC and PINP, which are markers of bone formation, did not differ among the three groups. ICTP and β-CTX levels mainly reflect the rate of bone resorption. The mean levels of β-CTX in the RA group were not significantly different from those in the OA group or healthy controls (*P* > 0.05). ICTP, which is a more sensitive marker, was higher in the RA group (5.52 ± 5.14 μg/L) than in the healthy controls (2.05 ± 0.71 μg/L, *P* < 0.001) or the OA group (2.45 ± 0.9 μg/L, *P* < 0.001). RA patients exhibited increased baseline plasma levels MMP3 (29.93 ± 18.57 ng/ml), which is a molecule responsible for degradation of the extracellular matrix, compared with the healthy controls (12.79 ± 8.17 ng/ml, *P* = 0.007) and the OA group (5.25 ± 1.95 ng/ml, *P* < 0.001). Levels of OPG were significantly reduced in RA patients compared with those of healthy controls (*P* < 0.001) or OA patients (*P* = 0.001). The concentrations of bone and inflammatory biomarkers are shown in Table 1.

**Table 1:** Levels of Bone turnover and inflammatory markers

	Control *n* = 12	OA *n* = 12	RA *n* = 14
TNF-α (pg/ml)	10.45 (8.05, 17.33)	14.4 (11.03, 20.58)	16.2 (12.53, 24.00)*
IL-1β (pg/ml)	<5	<5	<5
IL-6 (pg/ml)	2.00 (2.0, 2.38)	2.60 (2.0, 2.70)	9.70 (7.9, 71.95)^*,**^
IL-17 (pg/ml)	<15	<15	17.99 ± 1.81
ICTP (μg/L)	1.79 (1.59, 2.50)	2.22 (1.68, 3.11)	4.40 (2.98, 5.28)^*,**^
OC (ng/ml)	17.33 ± 4.43	14.25 ± 4.13	16.33 ± 8.81
β-CTX (ng/ml)	0.33 (0.25, 0.42)	0.47 (0.35, 0.56)*	0.28 (0.19, 0.43)
PINP (ng/ml)	48.07 ± 14.99	53.31 ± 16.73	48.89 ± 21.39
MMP-3 (ng/ml)	12.79 ± 8.17***	5.25 ± 1.95***	29.93 ± 18.57
OPG (pg/ml)	156.14 (151.48, 161.96)	96.45 (87.45, 145.37)*	73.79 (59.88, 89.75)

Continuous data are expressed as means ± standard deviation (SD) or medians (inter-quartile range, IQR) according to the data distribution

*Abbreviations*: *TNF-α* tumor necrosis factor-α; *IL-1β* interleukin-1β, *IL-6* interleukin-6, *IL-17* interleukin-17, *ICTP* collagen type I cross-linked telopeptide, *OC* osteocalcin, *PINP* procollagen type I N propeptide, *β-CTX* C-terminal telopeptide of type I collagen, *ICTP* collagen type I cross-linked telopeptide, *MMP3* matrix metalloproteinase 3, *OPG* osteoprotegerin

*P < 0.05 compared to healthy controls using the Mann–Whitney *U*-test

^**^ *P* < 0.05 compared to the OA group using the Mann–Whitney *U*-test

***P < 0.05 compared to the RA pretreatment group using Student’s *t*-test

### Correlations between DR2 expression on B cells and levels of bone turnover and inflammatory markers in the plasma

**Fig. 2:**
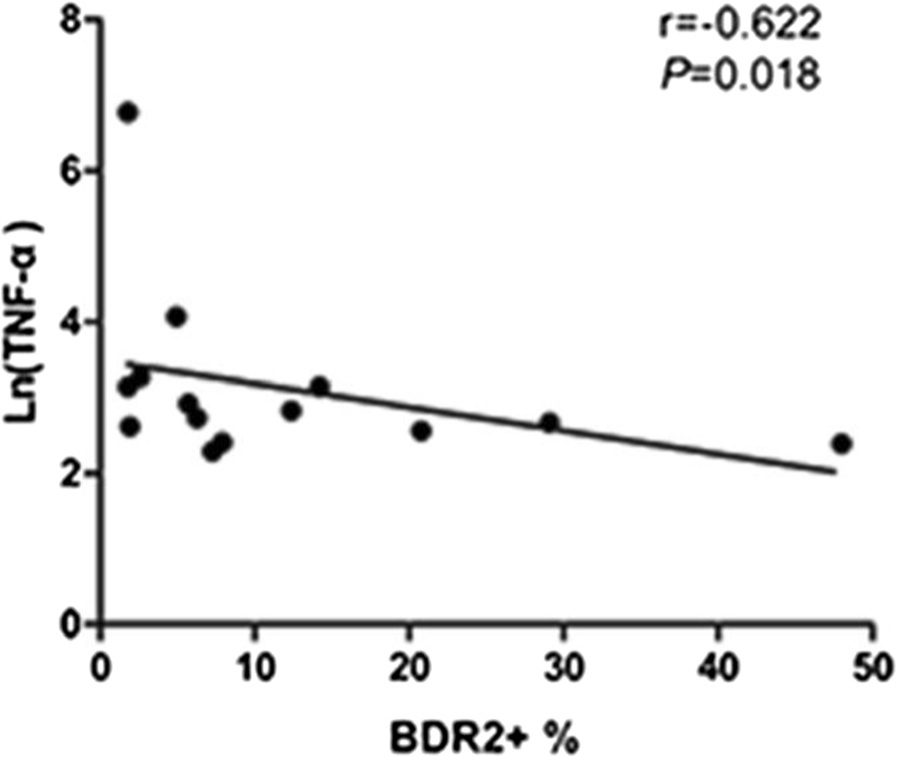
Spearman correlation analysis of DR2 expression and TNF-α level in RA patients

Associations between the frequencies of B cells expressing DR2 with levels of inflammatory and bone turnover markers were analyzed using Spearman correlation tests. We found that B cell DR2 expression had a negative correlation with TNF-α level in RA patients *(r* = −0.622, *P* = 0.018; Fig. 2). The secretions of TNF-α, IL-6 and IL17 have been demonstrated to cause synovial inflammation [15], However, DR2 was not associated with other cytokines except for TNF-α and bone turnover markers.

### DR2 expression on B cells in synovial tissues

**Fig. 3:**
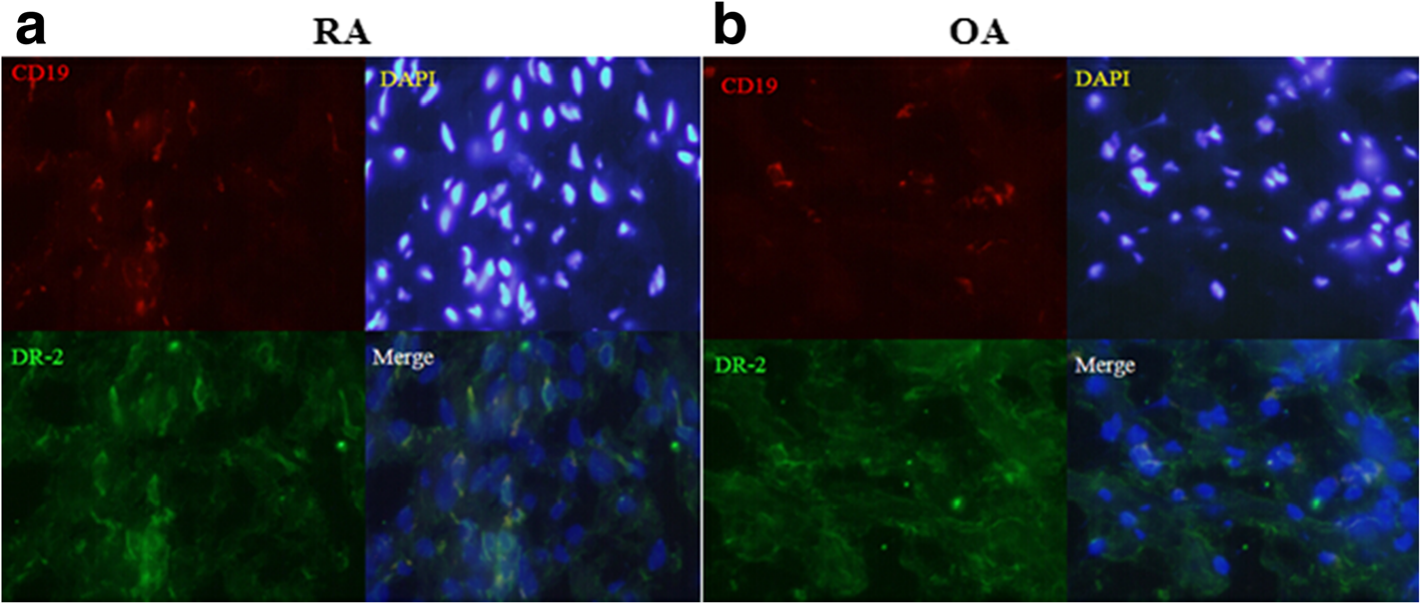
In-situ immunofluorescence stainings of B cell DR2 expressions in the synovial tissues. **a** and **b** Representative images of DR2 expression on B cells from RA (*N* = 4) and OA (*N* = 3) patients are shown. Sections were counterstained with DAPI (*blue*) to allow visualization on nuclei; magnification, 400×. DR2-positive cells are marked by an arrowhead

In the RA synovium, clusters of B cells could be observed at the sites of inflammation. Many B cells were found to express DR2 (Fig. 3a). Compared with RA synovial tissues, the inflammation was less severe and fewer B cells were found in whole sections from OA patients. Accordingly, few DR2^+^CD19^+^ B cells were detected in the OA patient samples (Fig. 3b). These distinct pathological findings were a consequence of the different immunopathologies involved in RA and OA.

## Discussion

Bone remodeling results from the dynamic balance of bone resorption and formation, and is subject to complex modes of regulation [16]. Imbalanced bone remodeling in RA leads to arthropathies, which include joint erosion and systemic osteoporosis. Dysregulated immune system and neuropeptides, such as dopamine, feed into the complex regulatory machinery that controls bone remodeling. One influence of immune signals can be to trigger disequilibrium of bone remodeling by affecting the differentiation of osteoclasts or the secretion of pro-inflammatory cytokines. Additionally, accumulating evidence indicates an intriguing role for the dopaminergic system in fine-tuning immune responses [17] and exerting direct effects on bone resorption [18]. Therefore, the fields of neuroimmunology and osteoimmunology are tightly integrated.

However, this composition of this intricate regulatory network remains obscure. In this present study, we investigated how the immune system, together with the dopaminergic system, can regulate bone metabolism, which determines disease manifestations and prognoses in RA patients. Previously, we showed that DR2 expression was negatively related to disease activity, and other DRs have been correlated with acute phase reactants or clinical manifestations. B cells are one of the most important drivers of immunopathologies, and pathological findings also support that B cells could contribute to both the initiation and perpetuation of pathogenic immune responses in RA [19]. Reports of the efficacy of B cell depletion in RA patients revitalized interest in the pathogenic role of B cells in RA. To further explore the relationship between DR2 expression level on B cells and bone metabolism, we analyzed levels of plasma markers of bone turnover and pro-inflammatory cytokines that might influence bone remodeling. Markers for bone turnover can be measured to detect differences in bone formation, resorption, further risk of fracture, and treatment responses in RA [20]. These biochemical markers show changes before bone mineral density changes that occur because of abnormal bone metabolism. Our findings indicated that MMP3 levels in RA patients were higher than those in OA patients and healthy controls (*P* = 0.007 and *P* < 0.001, respectively). ICTP, which is derived from MMP, could discriminate between RA patients from healthy individuals or OA patients, as well as MMP3. ICTP is a sensitive marker for periarticular bone resorption that is linked to the activity of MMPs in various cells, such as synoviocytes [21]. The RA patients which were in moderate to high disease activity had high levels of IL-17, IL-6 and TNF-α, suggesting that inflammation promotes the bone destruction. Our data also showed that MMP3 and ICTP represent effective biomarkers that reflect bone erosion associated with RA [22]. Although the correlation analyses indicated that DR2 expression on B cells from peripheral blood showed no association with bone turnovers, the previous report demonstrated the DR2 expressions on B cells were negatively correlated with disease activity. Therefore the focal expressions of DR2 on B cells found in synovial tissue might regulate the bone metabolism through affecting the inflammatory responses. TNF-α as a pro-inflammatory cytokine associated with bone metabolism can induce osteoclast activation and differentiation. TNF-α causes the osteoclast-induced bone destruction and can inhibit osteoblast differentiation and apoptosis [23]. TNF-α acts on chondrocytes to induce the synthesis of proteases, such as collagenase and matrix metalloproteinase, which can cause cartilage destruction [24]. Significant associations between treatment with TNF-α antagonists and the suppression of bone loss have also been described in many studies [25–27].

Previously, 10–25 % of infiltrating lymphocytes in RA synovial tissues have been reported to be B cells; however, this number might be an underestimate [28]. The synovial–bone interface in RA is an active site of germinal center formation, B-cell accumulation, plasma cell differentiation, *in situ* antibody production, and cytokine secretion [29]. Yeo et al. identified synovial fluid CD19^+^ B cells as the dominant producers of RANK-L at both the mRNA and protein levels. Compared to B cells in the peripheral blood, a higher proportion of these cells in the synovial fluid expressed RANK-L [30]. Our data showed that the distribution of B cells was mostly confined to clusters at sites of inflammation, as well as isolated cells or pairs of cells that infiltrated the synovium when viewed in the positive field. There were more infiltrating DR2^+^CD19^+^ B cells in the RA synovium than in the OA synovium. This finding laid the foundation for establishing the potential role of B cell expression of DR2 in modulating bone remodeling. However, in addition to B cells, a wide spectrum of cells expresses DRs, as we observed in our present study. Silvia et al. reported that DRs are also expressed by T cells, fibroblasts, and macrophages [7]. In 2003, a genotyping study from a group in Japan reported that the DR4 gene was significantly associated with bone mineral density and markers of bone resorption in men, establishing it as a candidate locus for predicting bone loss in Japanese individuals [8]. Subsequently, Martín et al. reported a significant positive association of urine dopamine levels with bone mass in the general population, which was not observed for other catecholamines [31]. The relationship between the dopaminergic system and bones was further explored in both animal models and *ex vivo* analyses. The D1-like receptor antagonist SCH23390 attenuated radiological joint destruction in mice with collagen-induced arthritis. Furthermore, the addition of SCH23390 inhibited osteoclastogenesis in a dose-dependent manner [10]. Another study by Nakano et al. characterized the effects of the dopaminergic system on the IL-6–IL-17 axis. Dopamine produced by DCs can induce IL-6-dependent secretion of IL-17, which is crucial for Th17 cell differentiation. SCH23390 can suppress the IL-6–Th17 axis, thereby alleviating cartilage destruction in a humanized RA/SCID mouse chimera model [7].

The present study has certain limitations. First, though our preliminary results demonstrated interest effects of dopamine receptor 2 expression on B cells on bone metabolism and TNF-α levels, these just indicated the phenomenon, the underlying mechanism remain to be elucidated in further study. The pending questions include that whether TNF was secretion from DRD2 + CD19+ B-cells and whether DRD2 expression on CD19+ B-cells is regulated by TNF stimulation. Second, the differences in DRD2 expression levels at each phase of B-cell differentiation should be done in the future to get better understand of the role of DRD2 expression on B-cells in the pathogenesy. Third, whether and how DR2 expression on B cells could affect T cells and other inflammatory cells, which might further promote the secretion of proinflammatory mediators, needs to be established. Finally, the limited patient cohort size should be expanded and more age and sex-matched controls would be enrolled in future studies.

## Conclusion

In conclusion, our study for the first time showed that infiltrating B cells express DR2 in RA synovial tissue, implicating that it might act to regulate bone remodeling. DR2 expression on B cells from the peripheral blood was negatively associated with levels of the proinflammatory cytokine TNF-α. This finding suggested that inflammation might be an important link between the immune system, neuropeptides, and the bones.

## Abbreviations

APC: Allophycocyanin
DAS: Disease activity score
DMARD: Disease modifying anti-rheumatic drug
DR: Dopamine receptor
ICTP: Collagen type I cross-linked telopeptide
MMP: Metalloproteinase
OA: Osteoarthritis
OC: Osteocalcin
OPG: Osteoprotegerin
PINP: Procollagen type I N propeptide
RA: Rheumatoid arthritis
TNF: Tumor necrosis factor
β-CTX: C-terminal telopeptide of type I collagen
